# Fragment-based virtual screening identifies a first-in-class preclinical drug candidate for Huntington’s disease

**DOI:** 10.1038/s41598-022-21900-2

**Published:** 2022-11-16

**Authors:** Simon Marius Galyan, Collin Y. Ewald, Xavier Jalencas, Shyam Masrani, Selin Meral, Jordi Mestres

**Affiliations:** 1 Galyan Bio Inc, 440 N Wolfe Road, Sunnyvale, CA 94085 USA; 2 Laboratory of Extracellular Matrix Regeneration, Department of Health Sciences and Technology, Institute of Translational Medicine, ETH Zürich, 8603 Schwerzenbach, Switzerland; 3 Chemotargets SL, Parc Científic de Barcelona, 08028 Barcelona, Catalonia Spain; 4 IMIM Hospital del Mar Medical Research Institute, Parc de Recerca Biomèdica de Barcelona (PRBB), 08003 Barcelona, Catalonia Spain; 5 Medicxi Ventures, 25 Great Pulteney St, London, W1F 9NH UK; 6 Biomedical Center Munich of the University of Munich, Großhaderner Str. 9, 82152 Planegg, Germany

**Keywords:** Chemical modification, Neurology

## Abstract

Currently, there are no therapies available to modify the disease progression of Huntington’s disease (HD). Recent clinical trial failures of antisense oligonucleotide candidates in HD have demonstrated the need for new therapeutic approaches. Here, we developed a novel in-silico fragment scanning approach across the surface of mutant huntingtin (mHTT) polyQ and predicted four hit compounds. Two rounds of compound analoging using a strategy of testing structurally similar compounds in an affinity assay rapidly identified GLYN122. In vitro, GLYN122 directly binds and reduces mHTT and induces autophagy in neurons. In vivo, our results confirm that GLYN122 can reduce mHTT in the cortex and striatum of the R/2 mouse model of Huntington’s disease and subsequently improve motor symptoms. Thus, the in-vivo pharmacology profile of GLYN122 is a potential new preclinical candidate for the treatment of HD.

## Introduction

Huntington’s Disease (HD) is an inherited neurodegenerative disease that results in the progressive decline of motor and cognitive functions and a range of behavioral and psychiatric disturbances^1^. The mean age of onset, i.e., the first appearance of symptoms, for HD is approximately 45 years. After the onset of symptoms, the debilitating progression of the disease proves invariably fatal, with a median survival time of 15 to 18 years^2^.

The gene responsible for HD was mapped to chromosome 4. Mutant huntingtin (mHTT) was characterized as containing a polymorphic trinucleotide repeat that is expanded in HD patients^3^. Although mHTT is ubiquitously expressed throughout the body, the most severe degenerative effects are seen in striatal neurons of the brain^4^. It has been demonstrated that the expansion in the polyQ stretch of the protein caused by the CAG repeat leads to mHTT having aberrant interaction with a multitude of other proteins of the cell^5^. But loss of function also plays a role in the pathogenesis of HD as it was demonstrated that huntingtin (Htt) is required for neuronal repair^6^.

Currently, there are no therapies available to mitigate the effects of HD. Therefore, there is a need for effective treatments for HD targeting mHTT as a strategy to treat the symptoms of the disease^7^.

Antisense oligonucleotides (ASOs) are complementary to Htt mRNA and can trigger their degradation after binding to Htt mRNA, subsequently reducing toxic mHTT levels. The ASO was regarded as a potential disease-modifying approach. Initial results from a 15-month open-label phase I/II study showed that 120 mg tominersen, an ASO, given every eight weeks, led to a 44% lowering of mHTT in the cerebrospinal fluid (CSF)^8^. But in phase III, tominersen fared worse in comparison to placebo on the primary motor outcome measures. The investigators of GENERATION-HD1 also reported drug-related increases in ventricular brain volume, implying more marked brain volume reduction in treated patients^9,10^. In the phase 1b clinical trial, neurofilament light protein (NfL) in CSF was elevated in some patients treated with 90 or 120 mg tominersen administered monthly for four months without accompanying MRI, indicating safety issues^8^. Effective therapies need to target deep brain structures, mainly the striatum in HD where motor symptoms originate in HD. ASOs as large molecules were reported to possess heterogenous penetration in deep brain structures, with marked less ASO accumulation in the striatum compared to other deep brain regions in male Sprague-Dawley Rats^11^.

A small molecule reaching deep brain regions more efficiently could be a better solution in HD. Various small molecules were developed for HD, including protein aggregation inhibitors^12^. However, HD pathology has been observed additionally to be caused by fragments, monomers, and oligomers of mHTT^13^. Only an approach which addresses all pathological forms of HD might be necessary to successfully to modify HD.

The availability of an X-ray crystal structure of an MW1 antibody binding to the polyQ tract of the mHTT protein opened an avenue to apply structure-based virtual approaches to prioritize small molecules likely to interact with mHTT^14^. The MW1 antibody was selected for binding robustly to the expanded polyQ repeat on Western blots of extracts from mutant Htt knock-in mouse^15^ and for its use in detection of mHTT in mHTT lowering clinical studies^16^. Here, we present the identification of a novel small-molecule hit binding to mHTT that was subsequently optimized to a first-in-class drug preclinical candidate for HD (GLYN122). A transgenic mouse model of HD was used to demonstrate the efficacy of our bioactive GLYN122 in suppressing motor deficits and mHTT aggregation, suggesting that GLYN122 might be a preclinical candidate drug.

## Results

### In-silico screening predicts small molecules that could bind mHTT

Our overall strategy was to combine in-silico with in-vitro screening to identify novel compounds that could reduce mHTT aggregation and then validate our lead compound in HD mouse models to ameliorate HD pathology.

**Figure 1:**
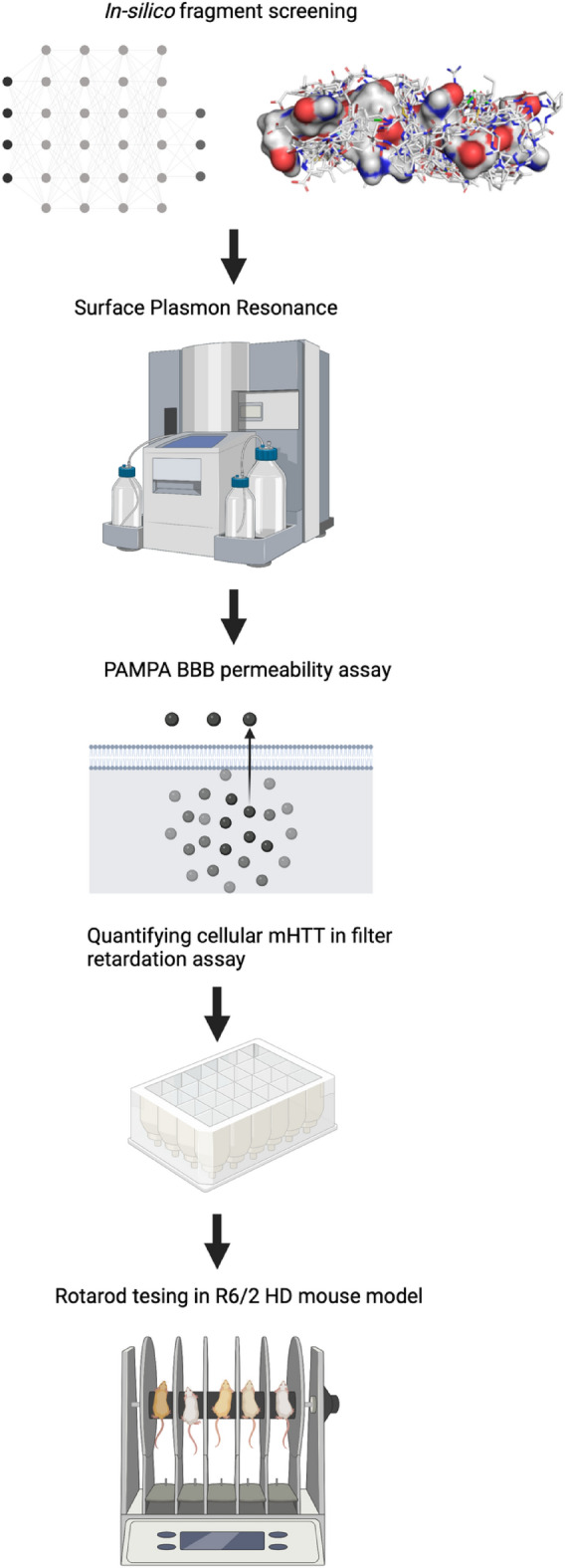
In the first step, we conducted an *in-silico*-based drug screening with a deep artificial neuronal network. Depicted are the selection of 67 small molecules docked on the surface of the polyQ tract peptide. 40 compounds were tested for their actual binding affinity to mutant huntingtin (mHTT) with Biacore, a system using surface plasmon resonance. Next, compounds were tested in an *in-vitro* mHTT assay and selected based on the ability of the compounds to lower mHTT and overcome an artificial blood–brain barrier in the PAMPA BBB permeability assay. Finally, we demonstrated in the R6/2 Huntington disease (HD) mouse model that the preclinical candidate GLYN122 can improve motor symptoms as measured with rotarod. Created with BioRender.com.

In the first step, a virtual protein surface scan was performed to identify fragment-binding environments from the Protein Data Bank (PDB) chemoisosteric to the polyQ tract surface (PDB entry: 2otu**)**^17,18^. Chemoisosterism is defined as the tolerance of the interaction between the same chemical fragment with different protein environments^18^. The virtual protein surface scan uses the selected surface to compare a database of 240,013 interacting pairs of chemical fragments and protein environments^17^ based on the identification of chemoisosteric protein environments^18^. When a protein environment was found to be similar to a surface area of the polyQ peptide, its associated chemical fragment was directly mapped onto the corresponding patch on the surface of the polyQ peptide. This resulted in a selection of 82 chemical fragments that were then used to filter a chemical supplier catalog of around 12 million commercially available compounds. Only 2937 compounds having at least two-thirds of their atoms substructural matched to any of the 82 chemical fragments passed the filter at this stage. They were then ranked by their predicted binding energy to the target structure using AutoDock^19^. A list of 67 compounds predicted to interact favorably with mHTT was generated from which a final set of commercially available 40 compounds was purchased from chemical vendors (Fig. 1).

### In-vitro validation of mHTT binding and drug optimization for reducing mHTT aggregation

**Figure 2:**
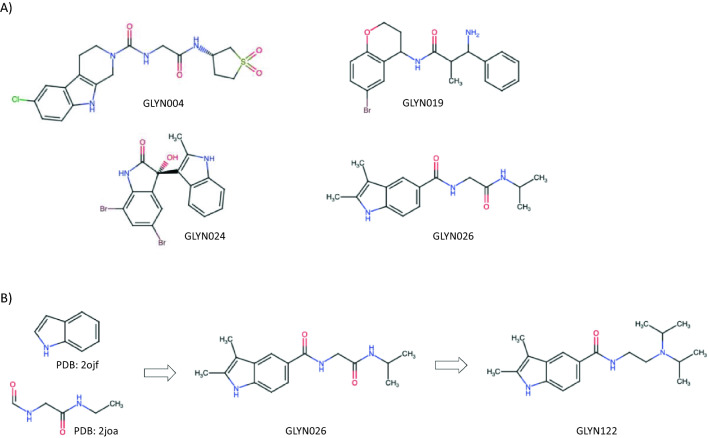
(**A**) Chemical structures of the 4 hits identified from the Biacore screening and (**B**) selection of the preclinical candidate (GLYN122) from the analogue expansion of one of the hit compounds (GLYN026) derived from two adjacent chemical fragments obtained from virtual protein surface scanning.

To determine whether the predicted small molecules interact with mHTT in vitro, we used Biacore as a label-free method to analyze the interaction between 40 compounds and the drug target (Supplementary Fig. S5, S6 and S7). A total of 4 hits were identified at this stage which showed a dose-response of binding, the most potent being GLYN026 (Fig. 2).

To identify derivates of GLYN026 with an improved binding capacity to mHTT, a similarity search was conducted on SciFinder (www.cas.org/) to select 60 commercially available analogs with Tanimoto similarity (TS) to any hits larger than 80% and fulfill Lipinski’s rule of five^20^. These 68 compounds were screened in the second Biacore screening round, and only two analogs of GLYN026 demonstrated a higher affinity towards Exon1 mHTT (Supplementary Fig. S8). These two second-round hits were subsequently used to identify 56 additional commercially available analogs based again on similarity criteria (TS > 80%), Lipinski’s rule of five, and low polarity (total polar surface area (TPSA) < 75 Å^2^) to increase the chances to penetrate the blood-brain barrier (BBB)^20^. In the third Biacore screening, we identified 20 compounds displaying improved affinity compared to the initial hit compound GLYN026. To validate these 20 GLYN026 analogues compounds with improved affinity, we tested them in the PAMPA BBB permeability assay^21^ and found that all 20 compounds were predicted in this assay to pass over the BBB. Thus, we had identified a hit compound GLYN026 and generated 20 analogs with improved binding to mHTT and the capability to pass the BBB.

Next, we selected 8 of these 20 compounds based on their best-presumed ability to penetrate the BBB for testing in a cell assay for their ability to clear mHTT. The effects of the eight hit compounds on mHTT aggregation were measured in the Htt14A2.6 PC12 line at five different dosages (concentrations) from 80 nM to 50 mM. Vehicle (DMSO) was used as controls. Cells with filter-trapped aggregates from cell lysates were quantified at both 24 and 48 h post-treatment time points by filter retardation assay. GLYN122 (Fig. 2) showed the highest potential to reduce mHTT. Thus, GLYN122 is our selected lead compound for further testing in vivo.

### GLYN122 penetrates the BBB in mice

**Figure 3:**
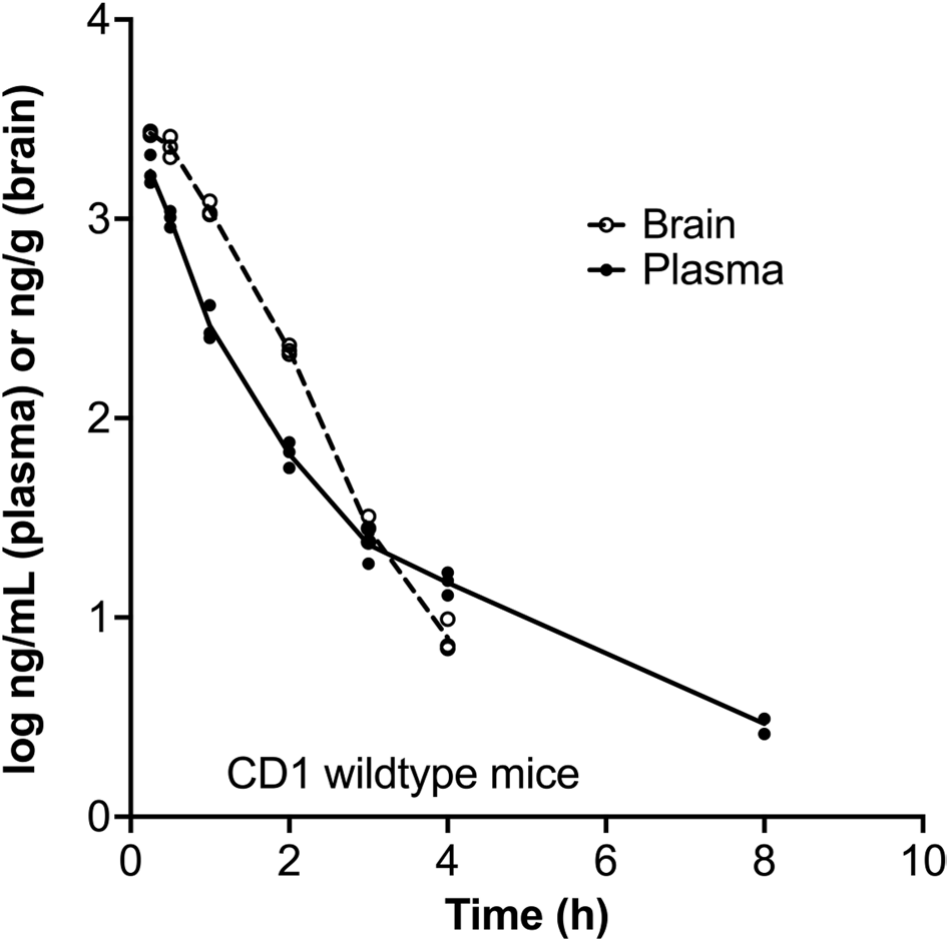
The concentration of GLYN122 that penetrated the blood–brain barrier of male CD1 mice (n = 3) treated with GLYN122 at a dose of 33.3 mg/kg body weight. Dashed line: brain, straight line: plasma.

To determine whether GLYN122 can actually pass the BBB in vivo, we measured the pharmacokinetics of GLYN122 in three two-months old male wildtype CD1 mice at several time points (Fig. 3). The tested dose was 33.3 mg/kg body weight injected intraperitoneally. Brain tissue was homogenized, and protein precipitated with acetonitrile. GLYN122 concentration was measured in plasma and brain with Ultra-High Performance Liquid Chromatography combined with Time-of-Flight Mass Spectrometry (UHPLC – TOF). This experiment showed that GLYN122 could penetrate the blood-brain barrier in vivo (Fig. 3). The half-life of GLYN122 in plasma and brain were 1.7 and 0.41 h, respectively. The maximum concentration C_max_ was 1753 ng/mL and 2692 ng/g in plasma and brain, respectively. The areas under the curve (AUC) were 1171 h*ng/mL and 2626 h*ng/g for plasma and brain, respectively. Thus, GLYN122 administration leads to detectable levels in the blood and in the target tissue, the brain. The pharmacokinetics suggests that GLYN122 might act as a hit-and-run compound, a suitable characteristic to avoid protentional long-term toxicities, indicating a preferential clinical profile.

### In-vivo studies of GLYN122 on HD pathologies

**Figure 4:**
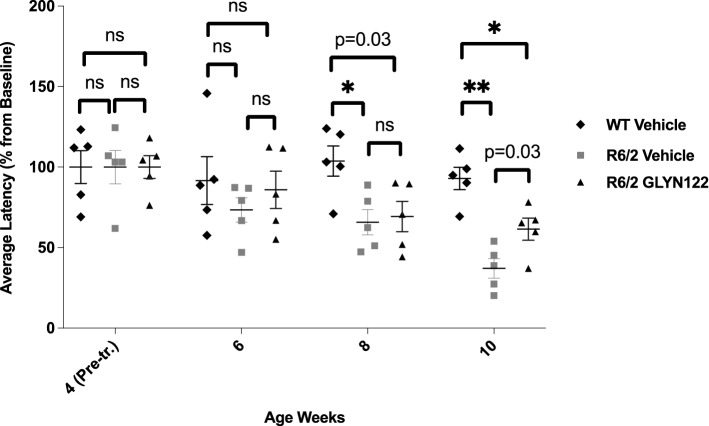
The effects of chronic administration of GLYN122 (33 mg/kg) on rotarod latency of female R6/2 mice at 4 to 10 weeks of age. Data are presented as percentage from 4-week pre-treatment baseline (mean + SEM) (WT Vehicle, n = 5; R6/2 Vehicle, n = 5; R6/2 GLYN122 33 mg/kg, n = 5). R6/2 GLYN122 33 mg/kg vs. R6/2 Vehicle. * *p* ≤ 0.01, ** *p* ≤ 0.001.

Next, we used the R6/2 HD transgenic mice as neuronal mHTT aggregates are associated with phenotypic onset in this model^22^. Among the key attributes of R6/2 are the extremely rapid progression of HD in the animals, a decline in rotarod latency, grip strength, and body weight, as well as the pronounced formation of mHTT inclusion bodies throughout the cortex and striatum^23^. We pre-treated WT (C57BL/6 J) and R6/2 with vehicle and R6/2 with GLYN122 for four weeks and assessed phenotypic changes at 6, 8, and 10 weeks during chronic administration. The rotarod latency of the vehicle-treated R6/2 mice was significantly decreased from 6 weeks onwards both in the current study and in the previously reported one^24^. Regarding the efficacy of GLYN122 treatment, the most notable finding in the present study was that the chronic treatment with GLYN122 (33 mg/kg, i.p., QD) significantly improved the rotarod performance of R6/2 mice at ten weeks of age mice (unpaired t-test, *p* < 0.05; Fig. 4).

The effects of chronic administration of GLYN122 (33 mg/kg) on grip strength of R6/2 mice showed no significant differences with the vehicle (unpaired t-test, *p* > 0.05) (Supplementary Fig. S1). Interestingly, bodyweight loss was significantly improved in R6/2 mice treated with GLYN122 at week 12 in comparison to untreated female R6/2 mice (unpaired t-test, *p* = 0.0003 for week 10) (Supplementary Fig. S2).

### GLYN122 lowers mHTT aggregation in vivo

**Figure 5:**
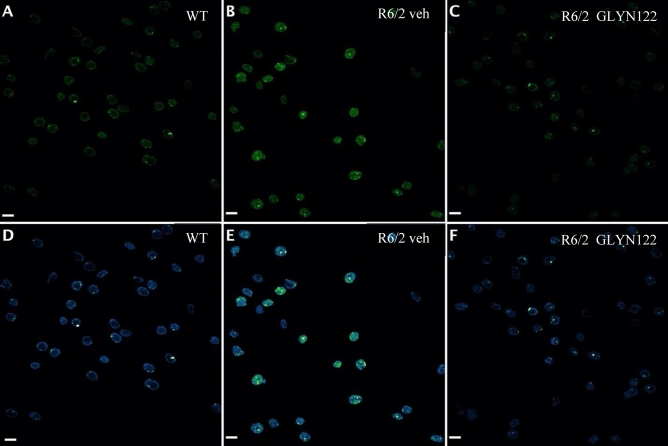
Confocal fluorescence images of the striatum region from WT vehicle (5A,5D), TG vehicle (5B,5E), and TG GLYN122 (5C,5F) samples labeled with EM48 mHTT antibody and detected with a fluorophore in the 488 nm channel. Grayscale images were pseudocolored with LUT “green” (top row) and “Green Fire Blue” (bottom row) in Fiji to visualize the gradient in signal intensities. For the latter LUT, increasing signal intensities are represented from blue to green to white. Overall, the strongest mHTT signal was visible in TG vehicles, while both WT vehicles and TG GLYN122 displayed decreased signals. Scale bars = 10 µm. WT wildtype, veh vehicle, TG transgenic. LUT Lookup Table.

**Figure 6:**
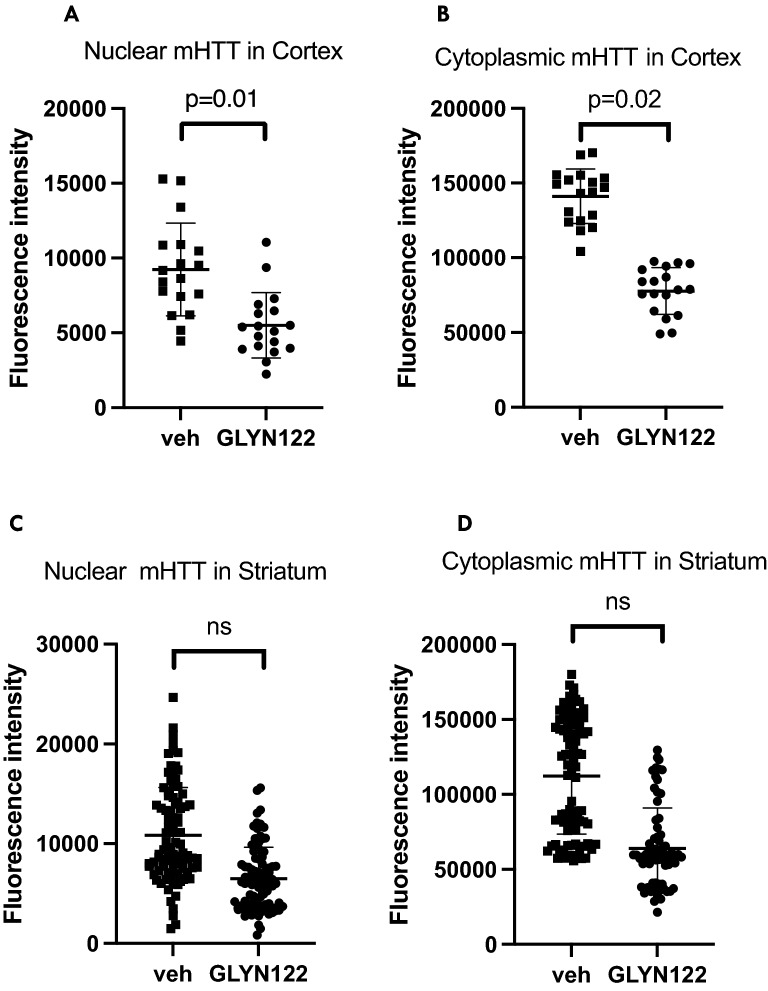
Diffuse mHTT reduction in the cortex (6A,6B) and striatum (6C,6D). Compared to a vehicle (veh), administration of GLYN122 reduced diffuse mHTT in the cortex by 40% in the nucleus (6A) and by 45% in the cytoplasm of treated TG mice (* p = 0.01 and p = 0.02, respectively). Likewise, GLYN122 reduced diffuse mHTT in the striatum by 40% in the nucleus (6C) and 43% in the cytoplasm (6D) compared to vehicle (veh) of treated TG mice (*p* = 0.14 and *p* = 0.06, respectively). The points on the graph represent different samples of the same animals.

To assess whether GLYN122 could prevent mHTT aggregation in vivo, confocal fluorescence imaging was used to distinguish between inclusion bodies (IB) and diffuse species of mHTT. Images were taken of the striatum to analyze mHTT aggregation within nuclei of R6/2 mice (Figs. 5A–6F). In the transgenic brain samples, large IBs were visible in many nuclei. Moreover, most IBs appeared to be surrounded by diffuse protein, visibly as a hazy signal in the nucleus. Consequently, this distinction between diffuse mHTT and IBs was implemented in the analysis macro to quantify the different species of mHTT within the striatum and cortex of R6/2 mice (Fig. 6). To this end, both an upper threshold of the diffuse protein fluorescence signal and an adjacent lower threshold of IB fluorescence intensity were set, and the images quantified appropriately. The antibody showed basal binding to endogenous mouse Htt with WT vehicle sample (Fig. 5A and D). Single bright foci of homogenous size were observed, which were thought to represent spontaneous self-aggregation of the antibody resulting from the absence of a specific antigen. By contrast, a substantial rise in total signal with transgenic (TG) vehicle sample relative to the results obtained with WT vehicle sample was observed (Fig. 5 and D). Bright, large, and more heterogeneous foci were described as IBs, with surrounding diffuse, oligomeric mHTT (see especially green-colored areas in lower depiction). Finally, Fig. 5C and F denote an apparent slump in mHTT signal when using a TG GLYN122 sample compared to the TG vehicle control. Both intensities of IBs and diffuse mHTT seemed to be substantially lowered in the treated sample. In general, the intensity level appeared to resemble the intensity of the WT control.

### Diffuse mHTT was reduced in the cortex of animals treated with GLYN122

Diffuse mHTT consists of monomers and oligomers. Diffuse forms of mHTT are highly toxic in comparison to mHTT aggregates^25^. In the cortex, diffuse nuclear mHTT was reduced by 40% in GLYN122 treated animals (n = 9) in comparison to vehicle-treated TG mice (*p* = 0.01) (Fig. 6A and B). No significant reduction of diffuse mHTT was measured in the striatum of female TG mice treated with GLYN122. In the striatum, we observed 40% diffuse mHTT reduction in the nucleus (*p* = 0.14) and 43% reduction in the cytoplasm in treated transgenic mice (*p* = 0.06) (Fig. 6C and D).

**Figure 7:**
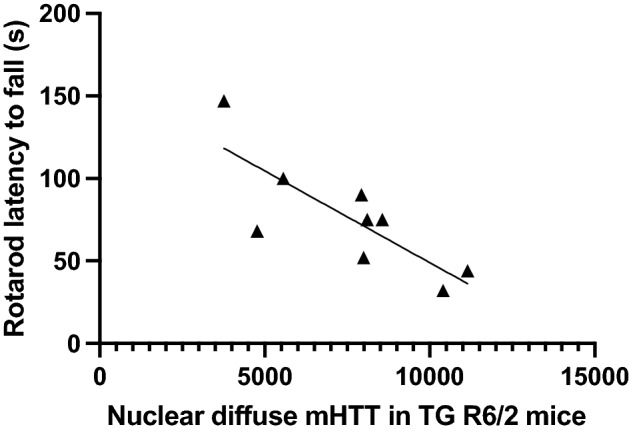
Correlation between rotarod and diffuse mHTT in R6/2 mice treated with GLYN122. Pearson r: − 0.8, p = 0.01, n = 9 (all transgenic female mice), confidence interval of r =  − 0.9561 to − 0.2896 for nuclear diffuse mHTT. TG, transgenic R6/2 mice; mHTT mutated huntingtin.

In the cortex of GLYN122 treated transgenic mice, on average 44% of nuclei were IB free and just on average 15% of nuclei in vehicle-treated transgenic mice. In the striatum, on average 47% in GLYN122 treated transgenic mice and on average 29% in vehicle-treated TG of nuclei were IB-free. Both results were statistically not significant (Supplementary Fig. S3). The reduction of diffuse mHTT correlated with motor symptom performance. Figure 7 shows that the motor symptoms strongly negatively correlate with the quantity of diffuse mHTT in the nuclei of the cortex (Pearson r: -0.8, *p* = 0.01, n = 9 (all transgenic mice), confidence interval of r =  − 0.9561 to − 0.2896 for diffuse nuclear mHTT).

### GLYN122 protective effects might be through enhanced autophagy

To gain insights into how GLYN112 might mechanistically work to reduce mHTT aggregation, we performed RNA sequencing on primary striatal mice neurons STHdh Q111/111 expressing mHTT were used (Supplementary data). Genes that are associated with autophagy were upregulated in mHTT expressing striatal neurons (STHdh Q111/111) treated with GLYN122 (Supplementary Fig. S4). Though, genes that are associated with autophagy were also upregulated in wildtype huntingtin expressing striatal neurons (STHdh Q7/7) treated with GLYN122 (Supplementary Fig. S5). This observation indicates that the autophagy inducing effect of GLYN122 might be related to a mHTT independent pathway which we are currently investigating. In summary, GLYN122 directly binds mHTT but, independently, also promotes other important processes, such as autophagy, suggesting GLYN122 might be a health-promoting drug candidate.

## Discussion

Recently, several HTT lowering approaches have failed in clinical trials. Here, we describe a small molecule that lowers mHTT, presumably through an increase in the degradation of mHTT. We discovered GLYN122 through an in-silico screening procedure and optimized the initial hits time cost-effectively. The oral availability of GLYN122 offers a clear delivery advantage over repeated intrathecal antisense oligonucleotides injections with more heterogenous mHTT lowering. As a small molecule, GLYN122 reduces mHTT in the cortex and striatum, which are crucial brain regions in the neuropathology of HD. The lowering of mHTT in the nuclei of neurons correlated with motor symptom improvements.

We found that GLYN122 could inhibit the progressive impairment of locomotor coordination in R/2 mice. But there was no improvement in grip strength. In a study, HD patients had reduced muscle strength by 50% on average than healthy matched controls^26^. Muscle weakness is a characteristic HD motor defect commonly attributed to central neurodegeneration. But hyperexcitable chloride and potassium channel dysfunction in muscle cells in HD patients was discovered, which could also contribute to muscle weakness^27^. Additionally, electron microscopy showed mitochondrial defects in myocytes in subjects with HD^28^. There is the support that mHTT may have detrimental effects on the skeletal muscles of R6/2 mice due to mHTT^29,30^. A similar transcriptional profile in skeletal muscles from R6/2 mice and HD patients has been identified^31^. As GLYN122 is two times higher in brain tissue in comparison to plasma, the cytoprotective effect of GLYN122 could be insufficient to cause an improvement in muscle strength. An optimization of GLYN122 could address this to increase the activity of the compound or changes in the dosing regimen.

GLYN122 showed a similar profile on motor symptoms as tolfenamic acid. Tolfenamic acid is a neuroprotective nonsteroidal anti-inflammatory drug. Like GLYN122, tolfenamic acid treatment did not reduce the weakening of muscle strength as captured by grip strength. But tolfenamic acid treatment significantly increased the latency in the rotarod test (*p* < 0.05).^32^ Additionally, tolfenamic acid decreased the expression of mutant huntingtin in the striatum of 20-weeks-old R6/1 mice^32^.

A small-molecule reaching deep brain regions more efficiently could be a better solution in HD. GLYN122 reduced mHTT in the striatum and cortex comparably, 40 and 45% in the nucleus, respectively. We assume that GLYN122 might penetrate the brain tissue more homogenously. But we still must demonstrate the CNS distribution of GLYN122 in future studies. However, the reduction in the striatum was not statistically significant. This could be due to the more pronounced pathology observed in the striatum despite abundant expression of mHTT in the entire brain. The pathological changes are most prominent in specific neuronal subpopulations affecting mainly the striatum and secondarily the cerebral cortex in patients with HD^33^.

The reduction of diffuse mHTT correlated with motor symptom performance in the R6/2 mice. Southwell and colleagues described that lowering mHTT in the CNS of Hu97/18 HD model mice is associated with a decrease in CSF mHTT^34^. They also observed that mHTT in CSF increased with HD stage^34^. Thus, mHTT lowering could be used as a biomarker for HD progression and surrogate for a clinical endpoint in a phase 1b/2a clinical trial of GLYN122 as it was done for the phase 1b clinical trials of tominersen^8^. Assays to measure mHTT in the CSF were developed and are available for clinical trials^35^.

We observed a normalization of weight gain in HD R6/2 mice treated with GLYN122 compared to untreated littermates. A previous study observed that at an early stage of the disease, subjects with HD had lower body weight in comparison to matched controls from the general population^36^. In another recent study, weight loss was associated with dysfunction of some regions of the brain in HD patients^37^. Thus, normalization of body weight gain might be an important secondary outcome parameter indicating a potential neuroprotective effect of GLYN122.

As an explorative study to identify the potential mode of action mechanisms of GLYN122, we measured gene expression in primary mouse neurons expressing mHTT (ST HDH Q111/111) and in primary mouse neurons expressing wild-tape huntingtin (ST HDH Q7/7) exposed to our preclinical candidate GLYN122. We observed an upregulation of genes involved in autophagy in both primary mouse neurons. It has been demonstrated that mHTT disrupts autophagy, contributing to the decreased clearance of aggregated proteins in HD^38^. Activation of autophagy using drug candidates successfully reversed HD-associated phenotypes in animal models^39,40^**.** As autophagy is necessary for neuronal survival and function^41^ GLYN122 might be of interest for other neurodegenerative diseases beyond Huntington disease. Small molecules regulating autophagy pathways have been demonstrated to possess therapeutic effects in various models of neurodegenerative diseases, such as Parkinson disease^42^, and Alzheimer’s disease^43^. However excessive activation of autophagy was associated with neuronal excitotoxicity^44^. Subsequently, identifying new and safe small molecule autophagy inducers is a highly sought therapeutic strategy for neurodegenerative diseases.

Our current findings in untreated R6/2 mice are well in line with previously reported results in studies produced at CRL Finland regarding the phenotype progression of the untreated R6/2 mice. Reviewing the data of the vehicle receiving transgenic mice in this study, the genotype difference in body weight was significant starting from 10 weeks, whereas, in a previous study, the bodyweight difference was effective from 9 weeks, although the weekly averages were close to the same level^24^. The rotarod latency of the R6/2 mice significantly decreased from 6 weeks onwards in the current study and in the previously reported one^24^**.**

The R6/2 model displays relevant disease-related phenotypes with limitations. The transgene comprises only the first exon of Htt. Consequently, the construct does not contain the HEAT domain, which is responsible for protein interactions of Htt^45^. We are planning a study in a knock-in mouse model of HD to verify our findings in the R6/2 mouse model. In these knock-in mouse models, the human HD mutation is directly inserted into the mouse HTT gene locus. Thus, the mutation is expressed in its correct genomic and protein context, representing HD pathology's genetic basis more precisely^46^.

## Conclusions

In summary, the present study results suggest that GLYN122 can attenuate motor deficits in R6/2 transgenic mice. GLYN122 reduced mHTT in the brain of R6/2 mice. Therefore, our data support GLYN122 as a preclinical candidate for the treatment of HD, which needs an enhancement of its potency to reduce the dose required to treat HD patients. Regarding the efficacy of the GLYN122 treatment, the most notable finding in the current study was that the chronic treatment with GLYN122 significantly improved the rotarod performance of female R6/2 mice at ten weeks of age.

## Methods

### Protein surface scanning

A crystal structure of the surface of an epitope on mHTT (PDB entry: 2otu) was described by means of surface feature points. The selected surface was then compared to a database of 240,013 interacting pairs of chemical fragments and protein environments extracted from the PDBbind database,^17^ using the ProSurfScan methodology based on the identification of chemoisosteric protein environments^18^. When a fragment-interacting surface environment present in PDBbind was found to be similar to a surface environment of the polyQ peptide, the chemical fragment of the former was directly mapped onto the latter. This resulted in a selection of 82 chemical fragments that covered a reasonable portion of the polyQ peptide interface to the antibody. These fragments were then used to filter a catalogue of around 12 million compounds from chemical vendors based on the assumption that compounds containing those chemical fragments will themselves favorably interact with the polyQ peptide. Accordingly, for each molecule in the catalogue, a substructure search was performed against the set of 82 chemical fragments. In the event that multiple fragments are detected as substructures of the catalogue molecule, the matching fragments were ordered by size and iteratively selected if compatible with all the already selected matches. Two chemical fragments were regarded as compatible if their volumes after the mapping were not overlapping. These criteria avoid selecting large compounds containing chemical fragments that were mapped onto the same region of the target surface. For a molecule to be selected, at least two thirds of their atoms are required to match any of the 82 fragments. A total of 2937 compounds passed the filter at this stage. This initial set of 2937 compounds was ranked by its predicted binding energy to the target structure using AutoDock^19^. To ensure a wide and diverse exploration of the polyQ peptide surface, compounds showing a surface overlap larger than 66% were filtered out and only the compound with the best predicted binding energy to a given surface region was kept. A list of 67 compounds predicted to interact favorably with the mHTT was generated. A final set of 49 compounds was purchased from chemical vendors.

### Protein expression and purification

Exon 1 of the HTT huntingtin (NCBI Gene ID 3064) was expressed as a fusion protein with (i) TRX (thioredoxin) only (MurTRX); (ii) 16Q (Mur16); and (iii) 46Q (Mur46). MurTRX, Mur16, and Mur46 were produced and purified by HIS-tag and size exclusion chromatography (SEC). Colonies were picked and 5 mL cultures were grown. Cells were induced at Optical Density (OD) 0.7–0.8 for four hours at 37 °C. 1 mL of the post-induction culture was spun down and the pellet resuspended in lysis buffer. This was spun down at 10,000 RPM for 10 min and the soluble fraction loaded on a 4–20% polyacrylamide gel (NuSep). An anti-His western blot was performed to detect protein expression. An ELISA assay was performed to confirm the expression and detection of the proteins. MurTRX, Mur16 and Mur46 are recognized by anti-His antibodies. Mur16 and Mur46 are recognized by the mHTT antibody 3B5H10 (Cat MABN821, Merck, Germany). Proteins were loaded onto IMAC resin (ThermoFisher Scientific HisPur) and eluted in 200 mM imidazole, purified by gel filtration FPLC (HiLoad superdex-200, 26/60; GE Life sciences) and concentrated using 3 kDa cut off Vivaspin 20 PES centrifugal concentrators (Sartorius AG). Proteins were biotinylated using a 1:0.5 molar ratio of EZ-link™ Sulfo-NHS-LC-LC-Biotin (ThermoFisher Scientific) and thoroughly dialysed against PBS prior to Biacore coupling.

### Neutravidin CM5 amine coupling immobilization

It was determined that the ligand density of streptavidin found on a standard SA Chip was not high enough for this project (~ 3,000 RU). It was decided to make a customized biotin capture chip using NeutrAvidin as the capture ligand as opposed to streptavidin. To obtain high enough ligand density on the sensor chip surface, NeutrAvidin (Thermoscientific Pierce, Waltham, MA USA) was immobilised onto all four sensor chip surfaces to a level of approximately 20,000 RU.

### Capture of the Mur ligands to NeutrAvidin

Biotinylated proteins were then diluted in HBS EP + running buffer and captured on the sensor chip surface. Mur46 was coupled to the sensor chip first as this is the major ligand of interest. After saturating FC3 with Mur46, target response levels for Mur16 and MurTRX were calculated solely based on their respective molecular weights. The respective response levels captured on the flow cells is summarized as follows: MurTRX – 4986RU with 15.13 kDa MW, Mur16 – 7762.2RU with 24.2 kDa MW, and Mur46 – 8996.2RU with 28.0 kDa MW. To investigate the binding properties of the coupled chip, 3B5H10 monoclonal antibody was diluted 1:600 and flowed over all four flow cells as a control (Supplementary Fig. S9 and Supplementary Table S3).

### Biacore screening

Briefly, compounds were diluted in 100% DMSO to a final concentration of 100 mM. These were diluted wherever possible to 1 mM, 0.1 mM, and 0.01 mM in 10% DMSO in PBS + running buffer. Where compounds were found to precipitate at 1 mM, compounds were prepared in 10% DMSO at twofold lower concentrations 0.5 mM, 0.05 mM and 0.005 mM. The entire assay was run in 10% DMSO in the Biacore T200 (GE, Little Chalfont in the United Kingdom) and an appropriate solvent correction window was selected for the assay. A multi cycle kinetics approach was used. Samples were run by identity, from the lowest to the highest concentration with one concentration per cycle. The assay was run with a 10 Hz data collection frequency in a multi (4–1, 3–1, 2–1) configuration and compounds were in contact with the ligands for 60 s with a flow rate of 50 μl/min and regeneration was carried out with 0.1 M glycine, pH 3.0 for 30 s. Blank samples (required for double referencing) were passed over the sensor surface every 21 cycles in triplicates, also every 21 cycles the positive MAb control (3B5H10) and solvent correction were run.

### PC12 cell assay

The Htt14A2.6 PC12 line was generated and propagated as described previously^47^. In brief, Htt14A2.6 was maintained in Dulbecco's modified eagle medium-GlutaMAX (Life Technologies, Grand Island, NY, USA) supplemented with horse serum (10%), heat-inactivated fetal bovine serum (5%), Pen Strip (1%), Zeocin (200 μg/ml), and G418 (50 μg/ml). For cell assays, cells in the log phase of growth were replated in 24-well culture plates around 50–60% confluency, grown overnight and induced for the expression of mHTTex1-GFP for 8 h with 2 μM ponasterone A (Santa Cruz Biotechnologies). Filter retardation was performed according to published methods^48^.

### R6/2 study

All animal experiments were carried out according to the National Institute of Health (NIH) guidelines for the care and use of laboratory animals, and approved by the National Animal Experiment Board, Finland. The animal facility at site is accredited by the Association for Assessment and Accreditation of Laboratory Animal Care (AAALAC), International. We complied with ARRIVE guidelines.

### General health status and humane end-points

Animals were monitored daily by laboratory personnel. The following humane end-points applied to all animals, unless otherwise mentioned in the experimental license granted by the National Ethics Committee. If the animal reached the humane end-points, it was euthanized. The animals’ welfare was assessed by observing the following signs: general appearance (dehydration, weight loss, abnormal posture, condition of skin and fur, signs of pain); ambulation (reluctance or difficulties to move); behavior (apathy, abnormal behavior); clinical signs (eating, drinking, urinating, defecating). In addition, the mouse was euthanized if the mouse was not able to right itself within 20 s when put on one side. If there was a deviation from normal, the animal was closely monitored and treated, when possible (e.g. hydration, analgesia, warming). As a general rule, the animal was monitored no longer than 24–48 h, after which the animal was euthanized, if its condition had not markedly improved.

### Husbandry

All mice were housed in groups of up to 5 per cage, in a temperature (22 ± 1 °C) and humidity (30–70%) controlled environment with a normal light–dark cycle (7:00—20:00 h light). All mice were housed in cages with clean bedding covering the ground that was changed as frequently as needed, at least once a week to provide the animals with dry bedding. This basic environment was enriched with the addition of a red mouse igloo (K3327), shredded paper and a wooden chewing stick. Food and water were available ad libitum to the mice in their home cages. The water spouts were fitted with extensions to allow mice to easily access from floor level. Each cage contained mice of only one treatment group. In each cage was included also a wildtype mouse to provide normal social stimulation to R6/2 mice.

### Breeding and weaning

10 female R6/2 mice and 5 female wildtype littermate control mice (F1 generation) were bred by Charles River Laboratories, Sulzfeld, Germany by mating (F0 generation) WT males (C57BL/6 J; systematically re-infused with pedigreed JAX mice, stock 000,664) with ovarian transferred (OT) TG females (JAX, stock 006,494). After weaning mice were sent from Germany to Charles River, Kuopio, Finland at an age of 3 weeks. Following genotyping and acclimation, the mice were enrolled in the study.

### Genotyping

Mice were ear marked at the age of 15—21 days and tail samples were collected at the same time for genotyping with PCR. Genotyping was performed at Charles River Discovery Services, Kuopio. DNA was isolated from tail or ear samples with Phire Animal Tissue Direct PCR-kit (Thermo Scientific, ref. F140WH) according to the kit’s instructions. Then 1 µl of DNA was multiplied in the PCR reaction with mouse specific (Gapdh) primers and human specific (Htt) primers. Primers sequences and final working concentrations are listed below. After the PCR, multiplied DNA was separated by agarose gel electrophoresis. The expected products were 272 bp (human specific product) and 372 bp (mouse specific product). Thus wildtype (WT) mouse has one 372 bp band while transgenic (TG) mouse has both the 272 bp and 372 bp bands.

Human specific 25 pmol/µl, (5′-3′): TCATCAGCTTTTCCAGGGTCGCCAT (SEQ ID NO: 8).

Human specific 25 pmol/µl, (5′-3′): CGCAGGCTAGGGCTGTCAATCATGCT (SEQ ID NO: 9).

Mouse specific 5 pmol/µl, (5′-3′): ACTCCACTCACGGCAAATTCAACGGCAC(SEQ ID NO: 10).

Mouse specific 5 pmol/µl, (5′-3′): GGTCATGAGCCCTTCCACAATGCCAAAG (SEQ ID NO: 11).

### Study design

10 female R6/2 mice and 10 female wildtype littermate control mice were bred at Charles River, Germany. Total of 20 female and male R6/2 mice and 10 female and male WT littermate control mice were used in the study. The mice were genotyped, and the R6/2 mice were divided into different treatment groups based on their litter and baseline body weight. The treatment with Vehicle or GLYN122 (33 mg/kg; 5 ml/kg, i.p. QD) was started at four weeks of age after the baseline behavioral tests. The experimental groups were: (i) Five wildtype mice treated with Vehicle (5 ml/kg, i.p., QD) starting at four weeks of age; (ii) Five R6/2 mice treated with Vehicle (5 ml/kg, i.p., QD) starting at four weeks of age; and (iii) Five R6/2 mice treated with GLYN122 (33 mg/kg; 5 ml/kg, i.p. QD) starting at four weeks of age. Animal care staff who administer treatments were unaware of allocation groups to ensure that all animals in the experiment were handled, monitored and treated in the same way.

### Body weight

Body weights were measured at three weeks of age and twice a week until the end of the study. Motor function testing using rotarod were commenced at four weeks (pre-treatment baseline) and continued at 6, 8 and 10 weeks of age, accompanied with grip strength at 4 (pre-treatment baseline), 10 and 12 weeks of age. At the end point of 12 weeks of age the mice were subjected to tissue collection.

### Motor function

The behavioral tests were conducted during the diurnal phase, between 8 a.m. and 5 p.m. The mice were transported to the behavioral test rooms from the animal housing rooms in their home cages. The mice were allowed to acclimate in the behavioral test room conditions at least for an hour before the tests. The behavioral tests were conducted under normal white light conditions.

### Rotarod test

Rotarod was performed at 4 (pre-treatment baseline), 6, 8 and 10 weeks of age. Each testing day included a training trial of 5 min at 4 RPM on the Rotarod apparatus (AccuScan Instruments, Columbus, USA). 30 min later, the animals were tested for 3 consecutive accelerating trials of 6 min with the speed changing from 0 to 40 RPM over 360 s and with an inter-trial interval of at least 30 min. The latency to fall from the rod was recorded. Mice remaining on the rod for more than 360 s were removed and their time scored as 360 s.

### Grip strength

Mice were tested at 4 (pre-treatment baseline), 10 and 12 weeks of age. Mice were taken to the experimental room and, one at a time, were placed on the grip strength apparatus (San Diego Instruments, San Diego, USA) in such a way that the animal grabbed a small mesh grip with its forepaws. The entire apparatus was placed on a table top for testing. Animals were lowered to the platform and then slowly pulled away from the handle by the tail until the animal released the handle. The equipment automatically measures the strength of the animal’s grip in grams. Five scores were recorded per animal in consecutive sequence, and the average of three best scores for each animal was used for the results. Mice were returned to their home cage after testing.

### End-point and tissue processing

Approximately one hour after the last dose the mice were terminally anesthetized with pentobarbital. Thereafter the mice were transcardially perfused with ice cold heparinized saline (Heparin 2.5 IU/ml) 25 ml)), followed by perfusion with ice cold 4% PFA (80 ml). The brains were fixed by immersion in 4% paraformaldehyde for minimum of 24 h after which brain samples were cryoprotected by 30% sucrose solution for 72 h after which the brain samples were frozen in liquid nitrogen. Frozen brain specimens were stored at − 80 °C.

### Preparation of immunohistology slides

Brain tissue collected within the animal study was prepared for IHC studies by Charles River staff. The brains were fixed by immersion in 4% paraformaldehyde for at least 24 h after which brain samples were cryoprotected by 30% sucrose solution for 72 h. Finally, the brain samples were flash-frozen in liquid nitrogen and stored at − 80 °C. The brain samples were cut using a microtome cryostat system, producing coronal brain tissue sections of 40 µm thickness. Those were mounted on individual adhesive-coated microscope glass slides with frosted ends.

### Staining

Goat anti-Rabbit IgG (H + L) Alexa Fluor 647 (1:500, ThermoFisher Invitrogen) was used as a secondary antibody for binding to CBP antibody (Supplementary Table S1). DAPI (Sigma-Aldrich) was used to identify the nuclei. Goat anti-Mouse IgG (H + L), Alexa Fluor 488 (1:500, A-11034, ThermoFisher Invitrogen) was used as a secondary antibody for binding to EM48 antibody. Mouse anti-human-mHTT (EM48, 1:500, Sigma-Aldrich), was used to stain huntingtin. Rabbit anti-CBP (1:100, Sigma-Aldrich) was used a primary antibody to stain CBP. The blocking buffer was freshly prepared and consisted of PBS (Sigma-Aldrich) with 5% normal goat serum (NGS), 0.2% BSA, 0.2% lysine and 0.2% glycine. Samples were covered with 750 µL of blocking buffer per sealing chamber and incubated at 4 °C for 24 h. Subsequently, on day two samples were washed three times 10 min each in PBS, before working dilutions of primary antibodies were applied in 750 µL primary antibody buffer per chamber. The primary buffer consisted of PBS with 2% BSA/0.3% Triton X-100 (Sigma-Aldrich) and 0.02% NaN3 as preservative agent. The samples were incubated with primary antibodies at 4 °C for 73 h. On day five, samples were washed like described previously and then incubated at 4 °C for 24 h with secondary antibody in 750 µL secondary antibody buffer at 1:500 working dilutions per chamber. The secondary buffer consisted of PBS with 3% NGS/0.3% Triton X-100/0.02% NaN3. On day six, samples were washed and then incubated with DAPI containing mounting medium Fluoroshield (Sigma-Aldrich), in order to counterstain the nuclei and preserve the fluorescence. Therefore, one drop of mounting medium (Dako) was added per tissue section and the sample carefully coverslipped avoiding introduction of air bubbles. The samples were stored for 24 h at room temperature shielded from light before being stored at 4 °C until imaging.

### Fluorescence imaging

Imaging sessions were performed on a confocal microscope system (Carl Zeiss Microscopy) equipped with a dual spinning disk unit (Yokogawa). All components of the imaging system were controlled via the ZEN 2 software suite (Carl Zeiss Microscopy). The laser lines used were 405 nm, 488 nm and 639 nm to excite DAPI or the respective fluorophores. The fluorescence images obtained of the immunofluorescence labelled tissue sections were quantified with the help of the “Image Processing and Analysis in Java” or short ImageJ software distributed under the GNU General Public License by the NIH^49^, i.e. the edition used was the Fiji distribution^50^. Nested t test was performed to calculate the significance amongst repeated measurements using GraphPad Prism software.

### Explorative mode of action study

STHdh 111/111 primary mouse neurons treated with GLYN122 at the indicated concentration for 48 h (Supplementary Table S2). Cells are washed with PBS, trypsinised, and gathered in a 15 ml falcon. After a short spin down for 3 min at 300 g, the cell pellet was washed with PBS once, before it was frozen down in liquid nitrogen. RNA was extracted with a standard protocol. The nCounter Mouse Neuropathology Panel (Nanostring, Seattle) was used for gene expression analysis according to the manufacturer’s protocols.

### Statistical analysis

Statistical analyses were performed using GraphPad Prism statistical software version 8.1.2 from 2019. Where not noted differently, significance levels were determined using unpaired Student’s t-test for all analyses, without assuming a consistent SD between sample populations. Where suitable, significance was reported as multiplicity adjusted p-value to account for multiple comparisons. The level for significance was set to alpha = 0.05 (95% confidence interval).

## Supplementary Information

Supplementary Information.

## Data Availability

The animal study datasets generated and/or analysed during the current study are available in the Harvard Dataverse repository, https://doi.org/10.7910/DVN/AYPCSD. All other data generated or analysed during this study are included in this published article [and its supplementary information files].
