# Supplementary material for: Fragment-based virtual screening identifies a first-in-class preclinical drug candidate for Huntington’s disease
Source: Sci Rep. 2022 Nov 16;12:19642. doi: 10.1038/s41598-022-21900-2 (PMC9668931; doi:10.1038/s41598-022-21900-2)
Supplement: Supplementary file 1 — Supplementary Information. [file 41598_2022_21900_MOESM1_ESM.docx]

Supplementary document

**Supplementary Figure S1**. The effects of chronic administration of GLYN122 (33 mg/kg) on grip strength of female R6/2 mice from 4-12 weeks of age. Data are presented as mean ± SEM (WT Vehicle, n = 5; R6/2 Vehicle, n = 5; R6/2 GLYN122 33 mg/kg, n = 5). p > 0.05 for all timepoints.


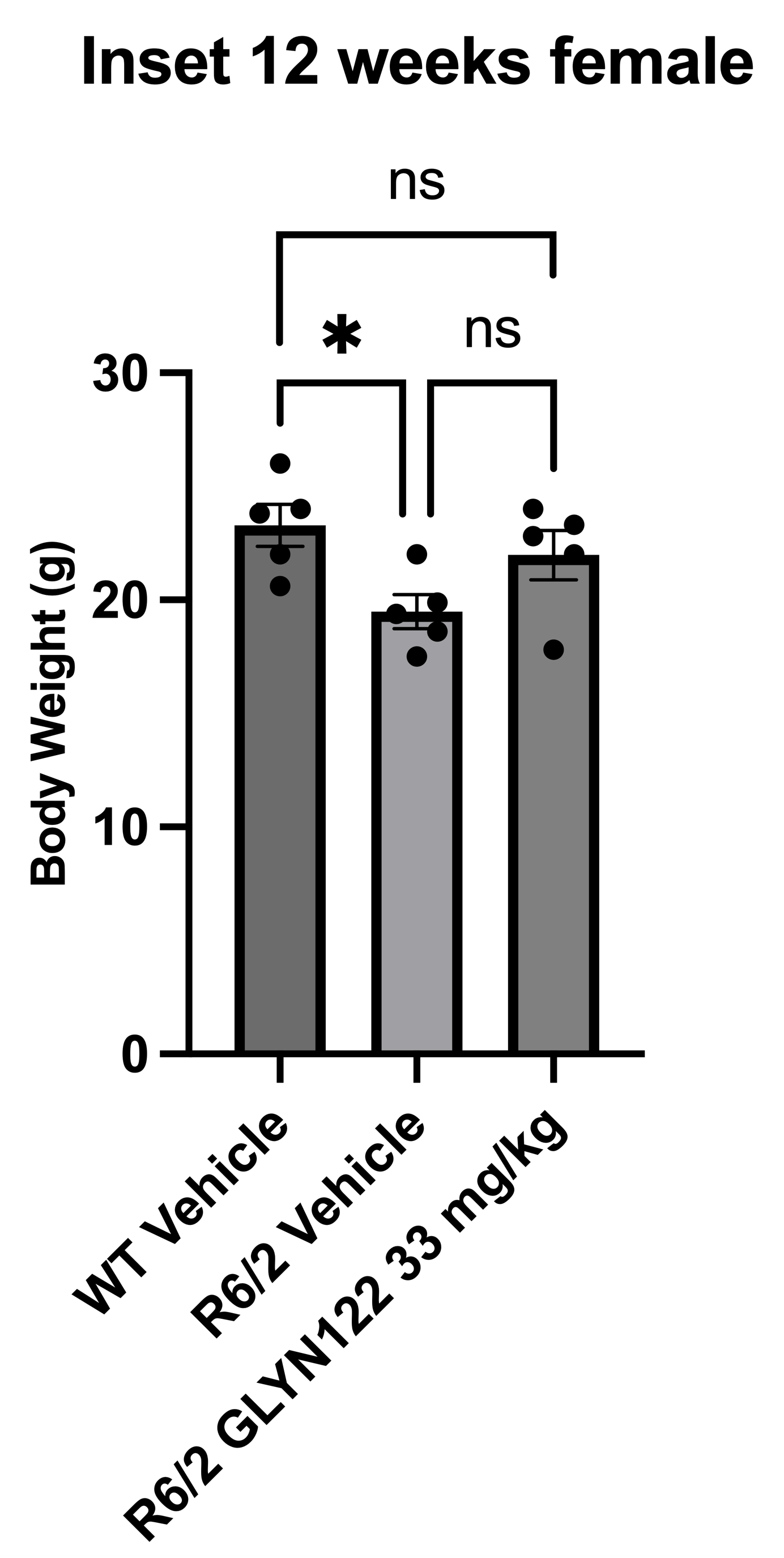

**Supplementary Figure S2**. The effects of chronic administration of GLYN122 (33 mg/kg) on body weight of female R6/2 mice aged 3 to 12 weeks. Data are presented as mean ± SEM (WT Vehicle, n = 5; R6/2 Vehicle, n = 5; R6/2 GLYN122 33mg/kg, n = 5). # p < 0.05, R6/2 GLYN122 33mg/kg vs. R6/2 Vehicle.


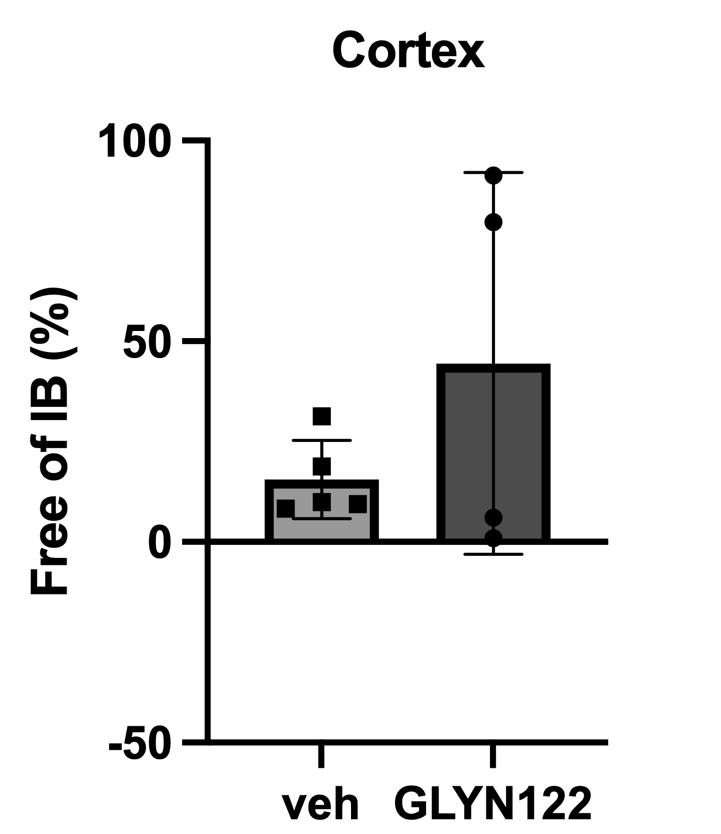


Supplementary Figure S3. In the cortex of GLYN122 treated TG 44% of nuclei were IB free and just 15% of nuclei in vehicle treated TG mice. In the striatum 47% in GLYN122 treated TG and 29% in vehicle treated TG of nuclei were IB free. Both results were statistically not significant.


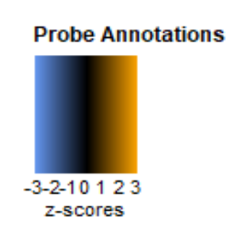

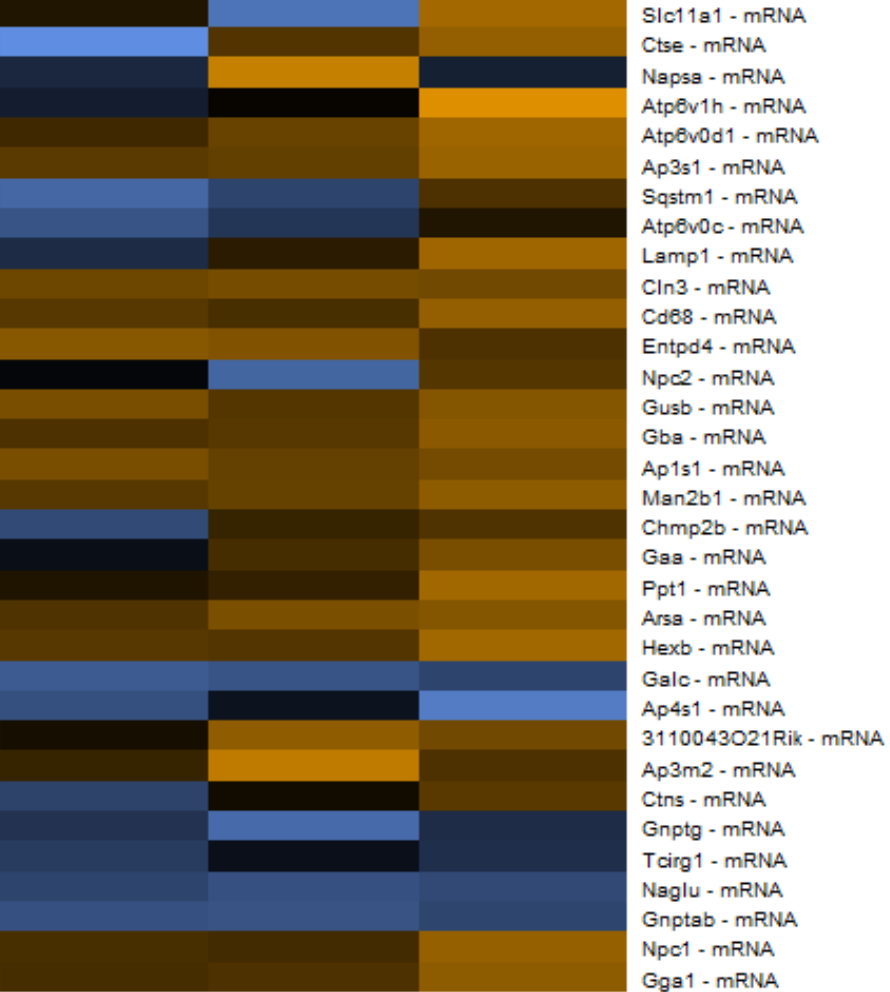


10 µM

NT

20 µM

Supplementary Figure S4 STHdh Q111/111 autophagy genes are upregulated in treated neurons. There is a dose response. NT no treatment, 10 µM of GLYN122 or 20 µM of GLYN122.

**Supplementary Table S1:** Antibodies Used in Immunohistochemistry Studies

**Antibodies**

Mouse anti-human-mHtt mab (clone mEM48) Merck MAB5374

Alexa Fluor 488 Goat anti-Mouse IgG (H+L) ThermoFisher Invitrogen A-11001

Alexa Fluor 647 Goat anti-Rabbit IgG (H+L) ThermoFisher Invitrogen A-21244

Alexa Fluor 647 Goat anti-Guinea Pig IgG (H+L) ThermoFisher Invitrogen A-21450

**Cell Culture**

*Cell lines used -* ST HDH Q111/111 (CH00095, Coriell Institute) striatal derived cell line from a knock in transgenic mouse containing homozygous HTT loci with a humanized Exon 1 with 111 polyglutamine repeats.

*Cell culture* - ST HDH Q111/111 (CH00095, Coriell Institute) striatal derived cell line was grown at 33°C in DMEM (Sigma-Aldrich), supplemented with 10% fetal bovine serum (FBS), 1% Penicillin-Streptomycin (ThermoFisher Scientific), and 0.4 mg/ml G418 (Geneticin; Invitrogen). ST HDH Q111/111 are mouse striatal cells with polyQ length of 111.

Treatment - Medium was removed and new DMEM (DMEM, high glucose, HEPES, no phenol red) medium, supplemented with 1% fetal bovine serum (FBS), 1% Penicillin-Streptomycin, containing 0µM, 10 µM or 20 µM compound was added. The cells were incubated at 33 °C for 48 hours.

**Supplementary Table S2**. STHdh 111/111 primary mouse neurons treated with GLYN122 at the indicated concentration for 72 h (hours).

| **SLOT** | **Cell Line** | **GLYN122 (µM)** | **Treatment time** |
| --- | --- | --- | --- |
| 1 | STHdh111/111 | 0 | **48 h** |
| 2 | STHdh111/111 | 10 | **48 h** |
| 3 | STHdh111/111 | 20 | **48 h** |

**Sequences of MurTRX, Mur16 and Mur46 Expression Vectors**

MurTRX Sequence Alignment

70HG41 MIAPILDEIADEYQGKLTVAKLNIDQNPGTAPKYGIRGIPTLLLFKNGEVAATKVGALSK

Designed MIAPILDEIADEYQGKLTVAKLNIDQNPGTAPKYGIRGIPTLLLFKNGEVAATKVGALSK

************************************************************

70HG41 GQLKEFLDANLAGSGSGERQHMDSPDLGTDDDDKGSGHHHHHH (SEQ ID NO: 2)

Designed GQLKEFLDANLAGSGSGERQHMDSPDLGTDDDDKGSGHHHHHH (SEQ ID NO: 3)

*******************************************

Mur16 Sequence Alignment:

sequenced MSDKIIHLTDDSFDTDVLKADGAILVDFWAEWCGPCKMIAPILDEIADEYQGKLTVAKLN

designed MSDKIIHLTDDSFDTDVLKADGAILVDFWAEWCGPCKMIAPILDEIADEYQGKLTVAKLN

************************************************************

sequenced IDQNPGTAPKYGIRGIPTLLLFKNGEVAATKVGALSKGQLKEFLDANLAGSGSGERQHMD

designed IDQNPGTAPKYGIRGIPTLLLFKNGEVAATKVGALSKGQLKEFLDANLAGSGSGERQHMD

************************************************************

sequenced SPDLGTDDDDKMATLEKLMKAFESLKSFQQQQQQQQQQQQQQQQPPPPPPPPPPPQLPQP

designed SPDLGTDDDDKMATLEKLMKAFESLKSFQQQQQQQQQQQQQQQQPPPPPPPPPPPQLPQP

************************************************************

sequenced PPQAQPLLPQPQPPPPPPPPPPGPAVAEEPLHRGSGHHHHHH (SEQ ID NO: 4)

designed PPQAQPLLPQPQPPPPPPPPPPGPAVAEEPLHRGSGHHHHHH (SEQ ID NO: 5)

******************************************

Mur46 Sequence Alignment:

sequenced XPSXNILFTLRRRYTMSDKIIHLTDDSFDTDVLKADGAILVDFWAEWCGPCKMIAPILDE

designed ---K-FCLTLRRRYTMSDKIIHLTDDSFDTDVLKADGAILVDFWAEWCGPCKMIAPILDE

: :****************************************************

sequenced IADEYQGKLTVAKLNIDQNPGTAPKYGIRGIPTLLLFKNGEVAATKVGALSKGQLKEFLD

designed IADEYQGKLTVAKLNIDQNPGTAPKYGIRGIPTLLLFKNGEVAATKVGALSKGQLKEFLD

************************************************************

sequenced ANLAGSGSGERQHMDSPDLGTDDDDKMATLEKLMKAFESLKSFQQQQQQQQQQQQQQQQQ

designed ANLAGSGSGERQHMDSPDLGTDDDDKMATLEKLMKAFESLKSFQQQQQQQQQQQQQQQQQ

************************************************************

sequenced QQQQQQQQQQQQQQQQQQQQQQQQQQQQQPPPPPPPPPPPQLPQPPPQAQPLLPQPQPPP

designed QQQQQQQQQQQQQQQQQQQQQQQQQQQQQPPPPPPPPPPPQLPQPPPQAQPLLPQPQPPP

************************************************************

sequenced PPPPPPPGPAVAEEPLHRGSGHHHHHH--AAALEHHHHHH-DPAANKARKEAELAAATAE

designed PPPPPPPGPAVAEEPLHRGSGHHHHHHAA-------------------------------

*************************** *

sequenced Q-LA-PLGASKRVLRGFLLKGGTISGLANGTRPVAAH-ARRVWWLRAA-PLHLPAP-RPL

designed ------------------------------------------------------------

* * * * *

sequenced LSLSSLPXXXXSPAFPVKL-IGGSL-XS (SEQ ID NO: 6)

designed ---------------------------- (SEQ ID NO: 7)

* *


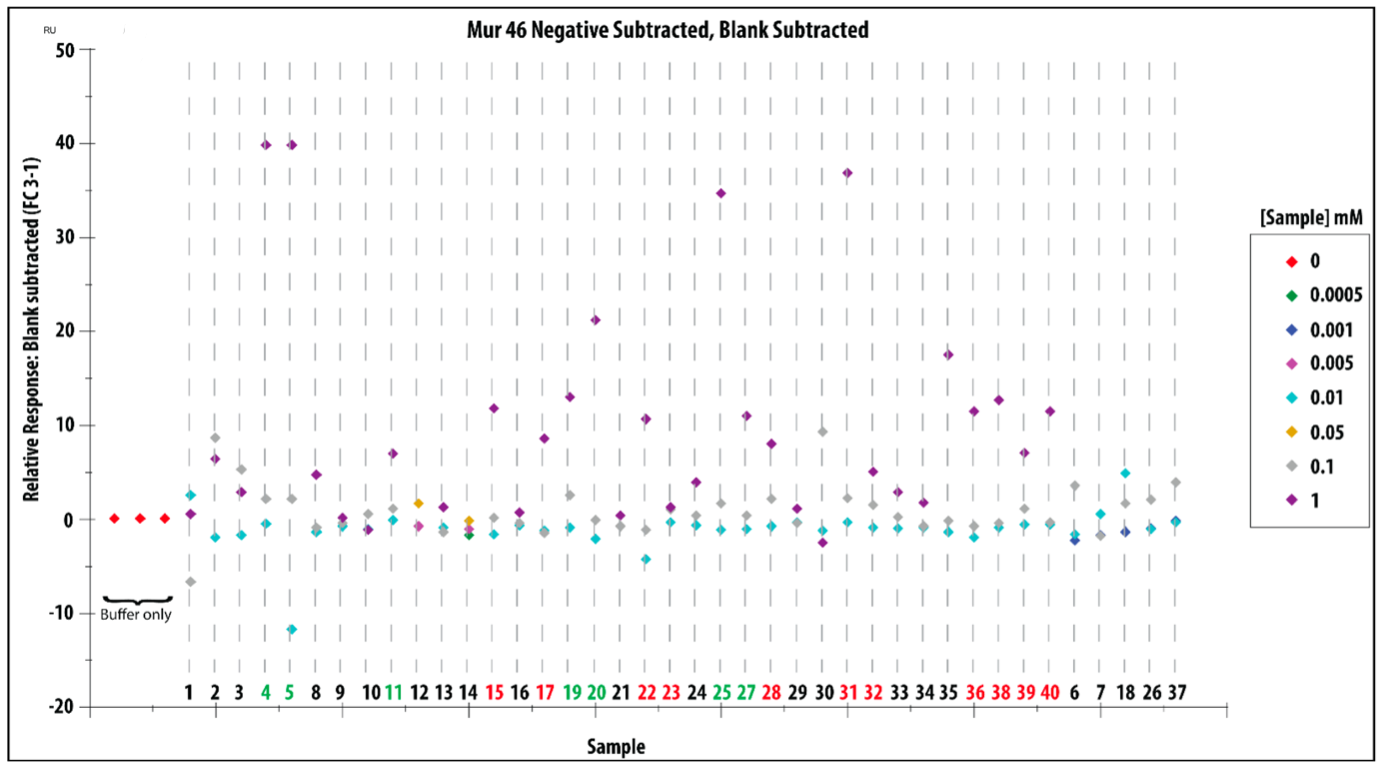


**Supplementary Figure S5**. The following compounds demonstrated higher binding in comparison to other compounds and additionally a dose-response with higher binding depended on concentration of the compounds: GLYN004 number 5, GLYN019 number 20, GYLN024 number 25 and GLYN026 number 27 in the figure.


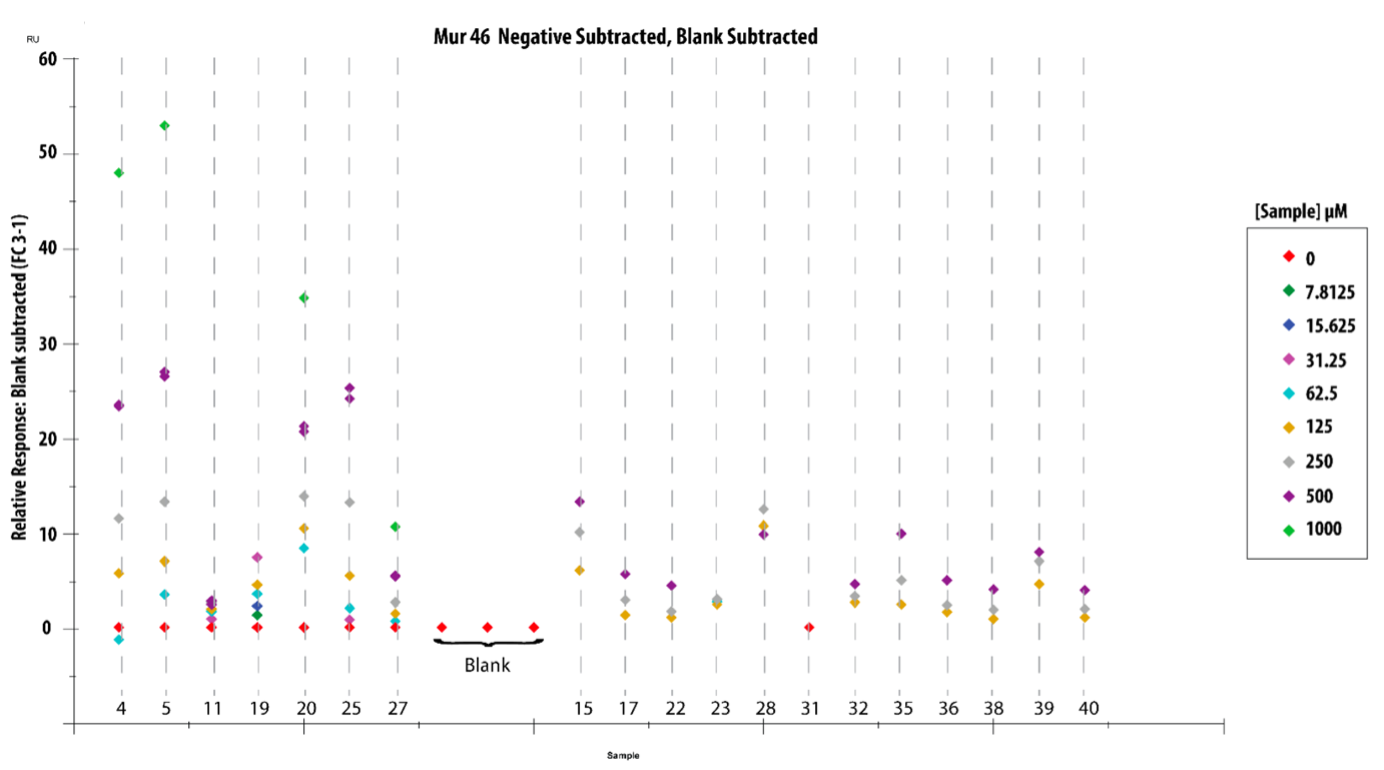


**Supplementary Figure S6**. Repeat of Biacore screening with different doses. Compounds are numbered the following GLYN004 is 5, GLYN019 is 20, GYLN024 is 25 and GLYN026 is 27.


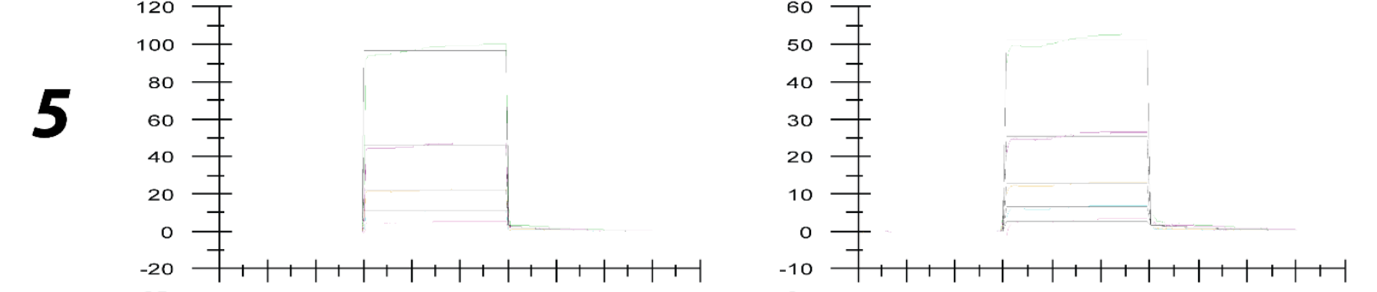


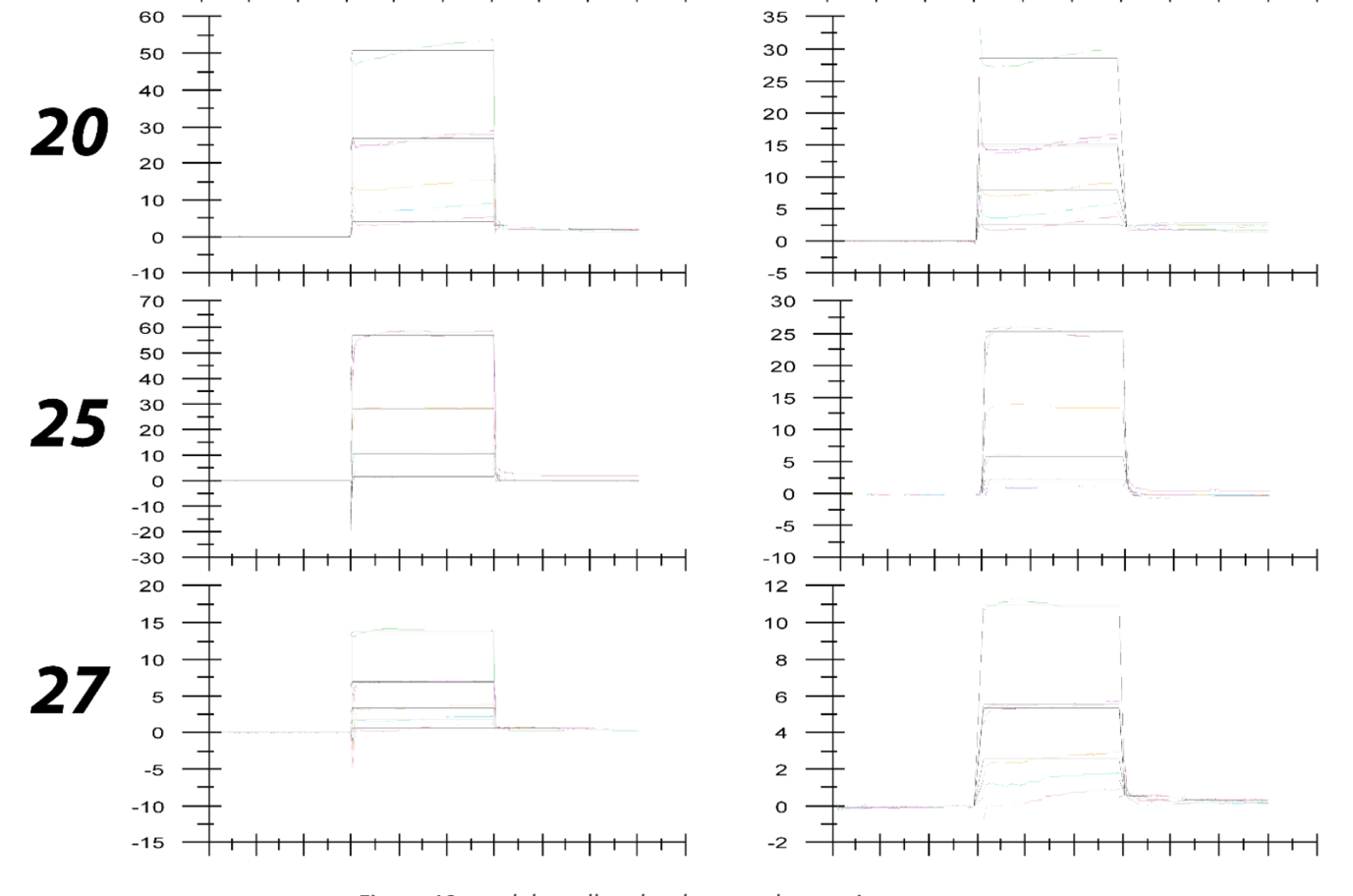


**Supplementary Figure S7**. Biacore sensorgrams of repeat screening of GLYN004 with the number 5, GLYN019 number 20, GYLN024 number 25 and GLYN026 number 27.


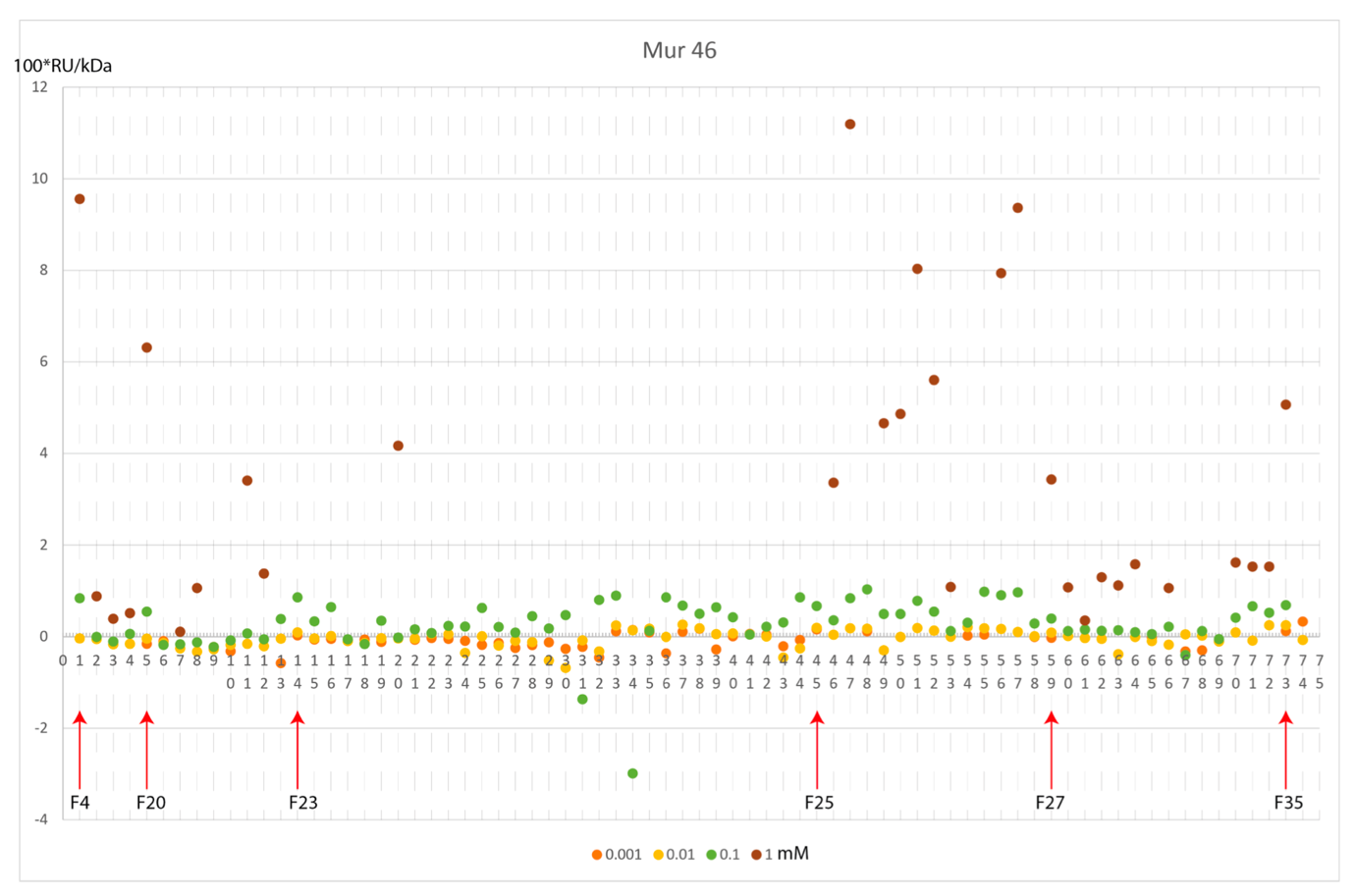


**Supplementary Figure S8**. Biacore sensorgrams of second round of analog screening. Mur 46 solvent was corrected, blank subtracted, molecular weight corrected dot plot. GLYN004 with the number F4, GLYN019 number F20, GYLN024 number F25 and GLYN026 number F27 the compounds are analogs following the original compounds marked out with a red arrow and the prefix F (forerunners) were from the first round which are numbered with an F for forerunners are analogs, respectively. Two analog compounds of GLYN026 have higher affinity at 0.1 mM in comparison to the original compound.


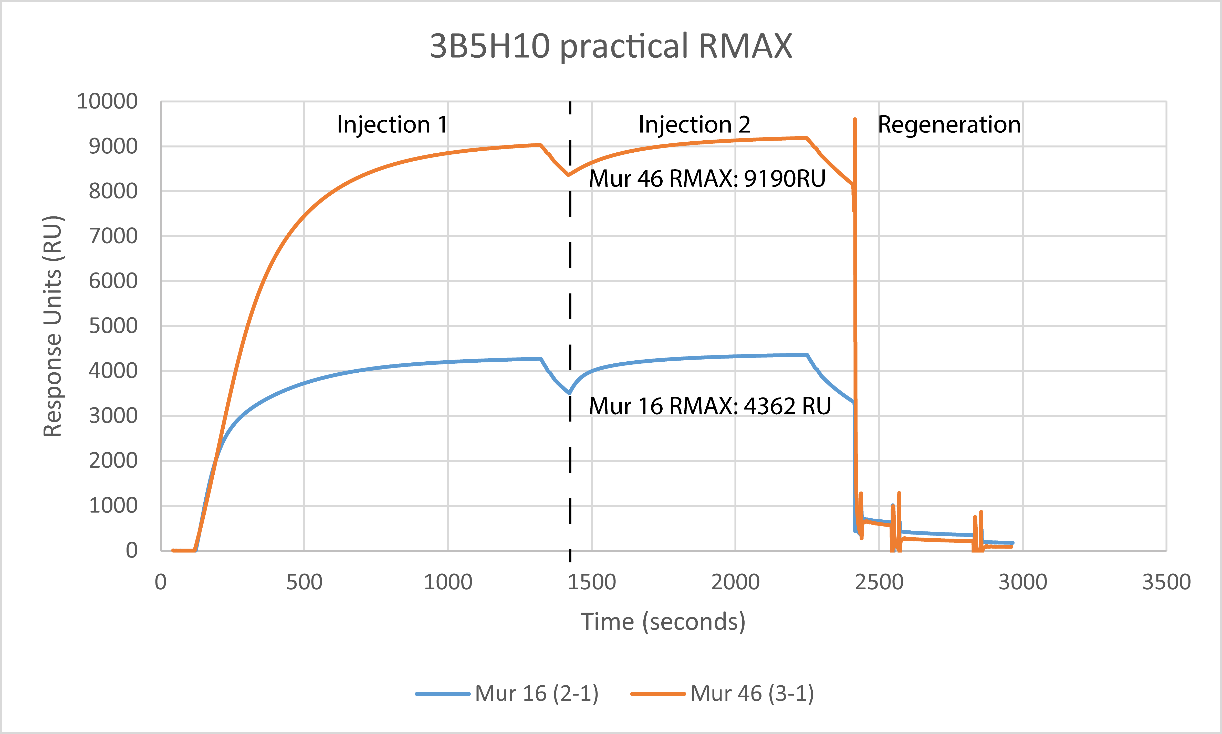


**Supplementary Figure S9** depicts the 3B5H10 saturation kinetics. To investigate the binding properties of the coupled chip, 3B5H10 monoclonal antibody was diluted 1:600 and flowed over all four flow cells to the point of saturation.

**Supplementary Table S3**. Theoretical compared with measured RMAX. The binding ability of the MAb to the ligand is lower than predicted.

| **Ligand** | **3B5H10 theoretical RMAX (RU)** | **3B5H10 measured RMAX (RU)** | **Calculated as % (measured/theoretical)** |
| --- | --- | --- | --- |
| MUR16 | 48,200 | 9,190 | 19.1 |
| MUR46 | 28,175 | 4,362 | 15.5 |
